# Protein kinase C-delta regulates HIV-1 replication at an early post-entry step in macrophages

**DOI:** 10.1186/1742-4690-9-37

**Published:** 2012-05-03

**Authors:** Xavier Contreras, Olfa Mzoughi, Fabrice Gaston, Matija B Peterlin, Elmostafa Bahraoui

**Affiliations:** 1 Université Paul Sabatier, EA 3038, 118 Route de Narbonne, Toulouse 31062, France; 2 INSERM, U1043, CPTP, CHU purpan, BP3028, Toulouse, Cedex3 31024, France; 3 CNRS, U5282, CPTP, CHU purpan, BP3028, Toulouse, Cedex3 31024, France; 4 Department of Medicine, University of California, San Francisco, CA 94143, USA; 5 CNRS UPR1142, IGH, Montpellier 34090, France

## Abstract

**Background:**

Macrophages, which are CD4 and CCR5 positive, can sustain HIV-1 replication for long periods of time. Thus, these cells play critical roles in the transmission, dissemination and persistence of viral infection. Of note, current antiviral therapies do not target macrophages efficiently. Previously, it was demonstrated that interactions between CCR5 and gp120 stimulate PKC. However, the PKC isozymes involved were not identified.

**Results:**

In this study, we identified PKC-delta as a major cellular cofactor for HIV-1 replication in macrophages. Indeed, PKC-delta was stimulated following the interaction between the virus and its target cell. Moreover, inhibition of PKC-delta blocked the replication of R5-tropic viruses in primary human macrophages. However, this inhibition did not have significant effects on receptor and co-receptor expression or fusion. Additionally, it did not affect the formation of the early reverse transcription product containing R/U5 sequences, but did inhibit the synthesis of subsequent cDNAs. Importantly, the inhibition of PKC-delta altered the redistribution of actin, a cellular cofactor whose requirement for the completion of reverse transcription was previously established. It also prevented the association of the reverse transcription complex with the cytoskeleton.

**Conclusion:**

This work highlights the importance of PKC-delta during early steps of the replicative cycle of HIV-1 in human macrophages.

## Background

Cells of the monocyte/macrophage lineage play a central role in HIV-1 infection and pathogenesis. In addition, macrophages play important roles for viral transmission and dissemination [1,2]. Indeed, the primary infection is initiated and carried out by macrophage-tropic viruses, which use, in addition to CD4, the CCR5 co-receptor. Macrophages are also one of the main reservoirs of HIV-1. This latter property is related to the lack of viral cytopathic effects in macrophages which ensures their survival when compared to infected CD4 positive lymphocytes [3-5]. Furthermore, current therapies that target HIV-1 replication are not as efficient in macrophages as they are in lymphocytes [6]. As a consequence, macrophages, in contrast to CD4 positive T cells, are not depleted during the course of HIV-1 infection. Thus, a better understanding of HIV-1 replication and the finding of efficient therapies for macrophages remain major challenges.

In addition to using CCR5 as the co-receptor for entry into its cellular targets, HIV-1 hijacks the underlying cellular machinery. Interactions between the viral gp120 envelope glycoprotein, CD4 receptor, and CCR5 co-receptor trigger a signaling cascade, which is comparable to that observed with their natural ligands. Initiated through the G-alpha proteins, these signals mobilize intracellular free calcium, translocate PKC, activate Pyk2, FAK. Erk1/2, Rho GTPases, and decrease levels of intracellular cAMP [7-12]. By facilitating the first steps of HIV-1 entry and trafficking in target cells, they play essential roles in the viral replicative cycle [9,13-19].

Among these pathways, PKC plays a critical role. In cells, where HIV-1 replicates efficiently, PKC must be activated. PKC isozymes (probably alpha and beta), which are activated by interactions between CCR5 and HIV-1, play a major role in the rearrangement of the actin cytoskeleton that is required for viral entry [9]. In addition to facilitating entry, via the phosphorylation of IκB (Inhibitor of NF-κB), PKC stimulates Nuclear Factor κB (NF-κB) [20-22]. NF-κB binds to the HIV-1 promoter and increases its transcription [23]. PKC also activates AP-1 and NF-AT [24,25] which also bind to the HIV-1 promoter. Moreover, PKC can phosphorylate a number of viral proteins such as p17Gag [26], Nef [27-29] and Rev [30], although the functional role(s) for their phosphorylation is poorly understood.

Eleven PKC isozymes have been described [31,32]. They have been classified depending mainly on their mechanism of action. They differ also in their subcellular localization and substrate specificity. Different types of cells express distinct PKC isozymes. Since PKC is triggered via CCR5, it is critical to determine which PKC isozymes are stimulated and their roles in the HIV-1 replicative cycle.

Of these, PKC-delta plays a central role in the differentiation of monocytes, which resist HIV-1 infection [33,34], to macrophages, which are permissive for infection [35,36]. Indeed, macrophage differentiation induced by monocyte colony stimulating factor (M-CSF) [37,38] or by PMA [39] depends on PKC-delta, which also activates NF-κB [38,40] and associates with vimentin in the cytoskeleton [41]. Additionally, the C2 domain of PKC-delta contains an actin-binding site. This binding could be involved in the redistribution of actin in neutrophils [42,43]. Thus, PKC-delta is a very attractive cellular cofactor for HIV-1 infection, particularly in macrophages. However, the expression of PKC-delta is not restricted to macrophages. Thus, effects of PKC-delta, which are addressed by this study, could be extrapolated to other cell types such as T lymphocytes, where the cytoskeleton also plays a critical role in the viral replicative cycle.

In this study, we characterized effects of PKC-delta on HIV-1 replication in human macrophages and demonstrated that it plays a critical role at an early step of infection.

## Results

### PKC-delta plays a major role in HIV-1 BaL replication in macrophages

**Figure 1:**
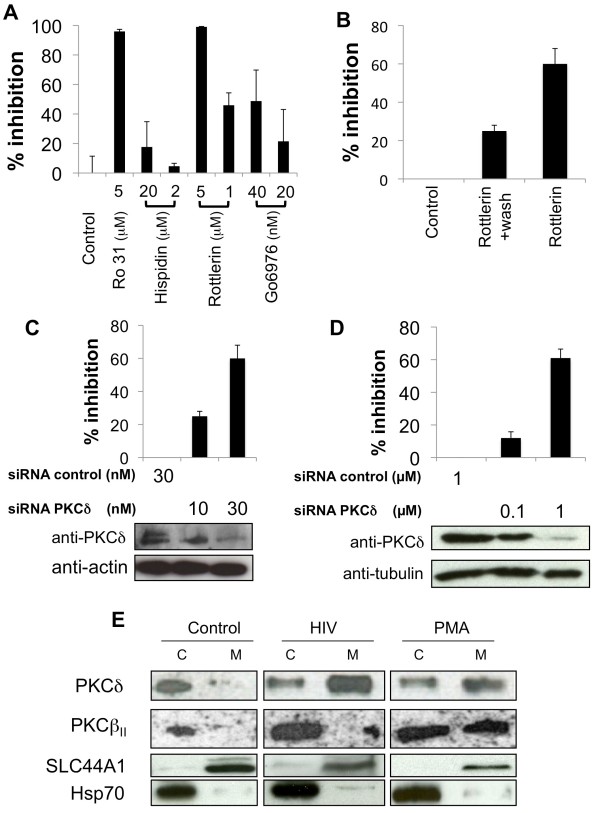
** PKC-delta is required for HIV-1 BaL replication in human macrophages.** (**A**) Macrophages (5 × 10^5^) were pretreated for 30 min with Ro31-8220, an inhibitor of all PKCs, or by selective inhibitors of certain PKC isozymes (rottlerin, PKC-delta inhibitor, Hispidin PKC-beta inhibitor and Gö6976, PKC-alpha, beta_I_, and mu inhibitor) at indicated concentrations. HIV-1 BaL infection (1 ng p24/ml) lasted 3 h. After 3 days culture supernatants were collected and viral replication assessed by the presence of p24 determined by ELISA. (**B**) 5x10^5^ macrophages were preincubated with rottlerin for 24 h and then cultured in rottlerin free medium for 24 h before infection to evaluate cytotoxic effects of rottlerin (rottlerin + wash). As positive control, the total inhibitory effect of rottlerin was evaluated as described in panel A. Error bars represent 3 experiments with one donor. Results are representative of experiments achieved in three different donors. (**C**) HeLa LTR-beta-gal cells or (**D**) macrophages were preincubated with siRNA against PKC-delta or scrambled siRNA for 2 days. Cells were then infected with HIV-1 BaL (1 ng p24) for 3 h and washed. After 48 h, cells were treated with X-gal and infection was assessed by counting beta-gal positive blue cells using microscopy (**C**) or by quantifying p24 in the supernatant (**D**). Western blot with anti PKC-delta antibodies is shown as the control. (**E**) Macrophages (5x10^6^) were infected with HIV-1 BaL (1 ng p24/ml) or stimulated by PMA for 30 minutes, and the activation of PKC isozymes was determined by analyzing PKC translocation to the membrane. Membrane bound (M) and cytoplasmic (**C**) proteins were extracted and separated by 10% SDS-PAGE. PKC-delta and beta_II_ isozymes were visualized by chemiluminescence using specific antibodies for each isozyme. Homogeneity of protein extracts was controlled by amido black staining of membranes. The purity of membrane and cytoplasmic fractions was controlled by Western blotting for SLC44A1 (membrane) and Hsp70 (cytoplasm). Results are representative of three independent experiments.

To determine the role of PKC in viral replication, macrophages were infected with the R5-tropic HIV-1 BaL in the presence or absence of chemical inhibitors of PKC. HIV-1 replication was assessed at day 3 post-infection using p24 ELISA (Figure 1A). Ro31-8220 (5 μM), which inhibits all PKC isozymes, decreased greatly (94%) viral replication (Figure 1A, lane 2). Interestingly, rottlerin (5 μM), a specific PKC-delta inhibitor [44], also blocked viral replication, whereas hispidin, a PKC-beta inhibitor, had little to no effect (Figure 1A, lanes 3 to 6). In addition, Go6976, which inhibits PKC-alpha, beta and gamma, had limited effects on viral replication (Figure 1A, lanes 7 and 8). These results suggest that PKC-delta plays an important role in HIV-1 infection of macrophages. Moreover, as assessed by trypan blue exclusion, rottlerin was not cytotoxic at these concentrations ( Additional file 1: Figure S1A); and HIV-1 BaL replication was similar in macrophages pre-treated or not with rottlerin (5 μM) for 24 h, and subsequently washed and cultured for an additional 24 h (Figure 1B and data not shown). Thus the effect of rottlerin is reversible. Strikingly, the preincubation of HeLa-CD4-CCR5-CXCR4 (HeLa-R5/X4) cells with increasing concentrations of siRNA [45] or antisense oligonucleotides targeting PKC-delta [46] inhibited viral replication by 62 and 85%, respectively, while control siRNA or sense oligonucleotides had little to no effect (Figure 1C and Additional file 1: Figure S1B). Indeed at these conditions, PKC-delta expression was suppressed strongly by siRNA or antisense oligonucleotides (Figure 1C, lower panels and Additional file 1: Figure S1C). Replication of X4-tropic viral strain HIV-1 VN44 was also inhibited in HeLa-R5/X4 pre-incubated with siRNA against PKC-delta ( Additional file 2: Figure S2). To further confirm the effects of the PKC-delta knockdown on viral replication, we infected primary human macrophages pre-incubated with siRNAs against PKC-delta with HIV-1 BaL. We observed a 60% inhibition of viral replication at conditions in which PKC-delta expression was reduced by siRNA (Figure 1D, lane 2). This inhibition was in agreement with decreased levels of PKC-delta (Figures 1C and 1D, lower panels). Altogether, these results demonstrate the importance of PKC-delta in the HIV-1 replicative cycle in macrophages.

Since PKC-delta plays an important role in viral replication, next, we sought to determine whether interactions between HIV-1 BaL and the target cell activate this isozyme. In unstimulated cells, PKC isoforms are localized to the cytoplasm. However, following their activation, they undergo conformational changes and translocate to the membrane. Taking this finding into account, we followed the activation of PKC-delta by its presence in cytoplasmic and membrane fractions in macrophages, which were pre-incubated with or without HIV-1 BaL. Figure 1E demonstrates that following 30 min incubation with HIV-1 BaL (1 ng p24), PKC-delta translocated to the membrane fraction of macrophages (Figure 1E, compare lanes 2 and 4). This activation was even stronger than that by PMA, a phorbol ester, which is widely used for the activation of PKC (Figure 1E, lanes 5 and 6). In contrast, in unstimulated cells, PKC-delta was present only in the cytoplasm (Figure 1E, compare lanes 1 and 2). On the contrary, PKC-beta_II_ did not translocate to the membrane after the incubation with viral particles, but only after macrophages were stimulation by PMA (Figure 1E, lanes 5 and 6). Taken together these results demonstrate a critical role for PKC-delta in viral replication. They also indicate that interactions between viral particles and target macrophages lead to its activation.

### Inhibition of PKC-delta restricts HIV-1 replication at a post-entry step

**Figure 2:**
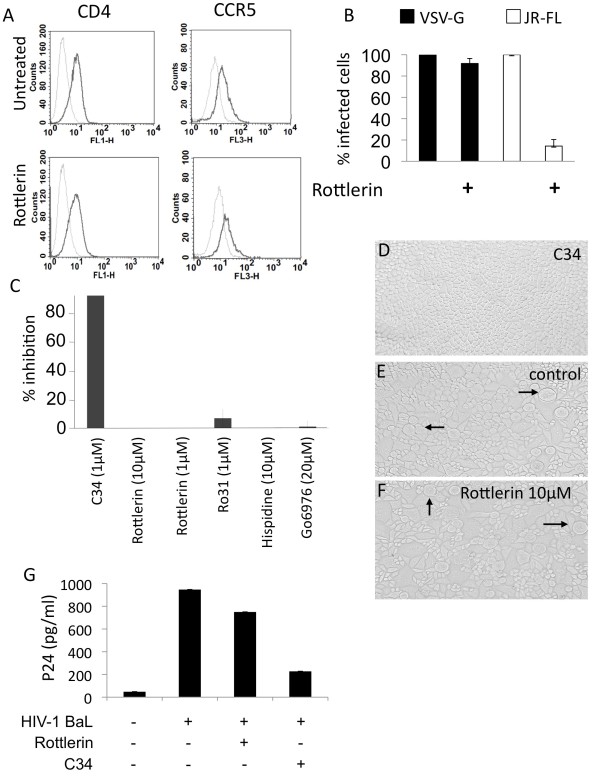
** Effects of rottlerin on viral entry.** Macrophages (5 × 10^5^/ml) were untreated or treated with rottlerin (5 μM) (**A**) for 30 minutes or (**B**) 2hours. (**A**) Cells were then stained for CD4 or CCR5 and analyzed by FACS. Dashed lines correspond to unstained cells, solid lines to specific staining. (**B**) Cells were then transduced with equal MOI of VSV-G- or JR-FL-pseudotyped vectors carrying a GFP reporter plasmid. Two days later, GFP-positive cells were counted using microscopy. Results are represented as relative levels of infection, with infected control cells set to 100%. Error bars are from triplicates of a single experiment. Results are representative of 3 independent experiments. (**C**) Cell fusion was measured by syncytia formation in a co-culture of cells expressing CD4 and CCR5 or gp120 and gp41, in the presence or absence of PKC inhibitors rottlerin, hispidin, Ro318220, Co6976, or the fusion inhibitor C34 as the control. (**D**), (**E**) and (**F**) Phase contrast microscopy images of syncytia in a coculture of cells expressing either CD4 and CCR5 or gp120 and gp41, in the presence of C34 (**D**), rotllerin 10 μM (**F**) or without inhibitors (**E**). Arrows indicate syncytium formation. (**G**) Macrophages were pretreated or not with rottlerin or C34 fusion inhibitor for 30 minutes and infected with HIV-1 BaL for 3 h, washed and treated with trypsin. After extensive washing, cells were lysed and p24 quantified using p24 ELISA.

To determine the role(s) of PKC-delta on viral entry, we first measured the expression of cell surface markers required for interactions between HIV-1 and macrophages, i.e. CD4 and CCR5, by flow cytometry (Figure 2A). Preincubation of macrophages with rottlerin (5 μM) had no significant effect on the expression of CD4 and CCR5. This result suggests that PKC-delta does not affect the expression of HIV-1 receptor or co-receptor. Next, macrophages were transduced in the presence or absence of rottlerin with lentiviral vectors coding for GFP (Green Fluorescent Protein) and pseudotyped with the envelope glycoprotein of the M-tropic HIV-1 JR-FL or the VSV-G protein. In addition to its wide tropism, the G protein of VSV mediates virus entry by endocytosis in a pH dependent manner. This situation is unlike that with the HIV-1 envelope glycoprotein, which mediates virus entry via a pH-independent mechanism. Cells transduced by these vectors were analyzed for the expression of the GFP gene. Figure 2B demonstrates that macrophages were transduced successfully by both vectors. When these experiments were performed in the presence of rottlerin (5 μM) (Figure 2B), the number of GFP-positive cells was similar to that found with VSV-G pseudotyped vectors in the absence of this inhibitor. In contrast, when examined under the same conditions, this number was strongly reduced for HIV-1 JR-FL pseudotyped vectors (90% inhibition). Thus, the inhibition of PKC-delta has a strong effect on HIV-1 JR-FL, but not VSV-G pseudotyped viral particles. These results demonstrate that the mode of entry determines the requirement for PKC-delta. Indeed, both vectors have similar mechanism by which their RNA is reverse transcribed, integrated and expressed, but differ in their mechanism of entry. Previous studies suggested already that, after entry via endocytosis, the viral genome in the reverse transcription complex is released in close proximity to the nucleus and thus does not require migration across regions of the cell such as the actin cortical mesh [47]. Thus, both the mode of entry and early post-entry steps are different in HIV-1 JR-FL and VSV-G pseudotyped lentiviral vectors. To discriminate between these two possibilities, we examined the formation of syncytia between HeLa-R5/X4 and HeLa-gp120/gp41, which express the envelope from the R5 tropic HIV-1 ADA. Under these conditions, rottlerin and other PKC inhibitors did not block the fusion of membranes (Figures 2C-F). To determine effects of PKC-delta inhibition on viral entry, we also pretreated macrophages first with rottlerin and then incubated them with HIV-1BaL for additional 3 hours at 37°C. To remove adsorbed viruses, cells were treated with trypsin. We used levels of intracellular p24 (measured by ELISA) as a marker of virus entry. Indeed, similar levels of p24 were found in cells treated or not with rottlerin (Figure 2G). As a control, to ensure that levels of p24 correspond to intracellular antigen and not to adsorbed viruses after trypsin digestion, we used a known inhibitor of fusion, the C34 peptide [48]. In its presence, the virus continues to bind to its receptors, but it becomes unable to induce membrane fusion. As expected, levels of p24 dropped strongly in the presence of the C34 peptide, confirming the specificity of this assay (Figure 2G). Taken together, these results indicate that blocking PKC-delta does not interfere with virus entry and further suggest that this inhibition occurs at an early step in the viral replicative cycle.

### Inhibition of PKC-delta affects an early step of reverse transcription

To determine effects of inhibiting PKC-delta on transcription, HeLa-R5/X4 cells, which contain an integrated LTR-beta-galactosidase reporter gene, were incubated in presence of GST-Tat ( Additional file 3: Figure S3). The addition of rottlerin had only small effects on GST-Tat-induced transactivation of the HIV-1 LTR. Similarly, transduction of macrophages with VSV-G pseudotyped lentiviral vectors encoding GFP under the control of HIV-1 LTR led to equivalent levels of GFP expression in the presence or absence of this inhibitor (Figure 2B). These results suggest that inhibiting PKC-delta does not affect HIV-1 transcription and gene expression.

**Figure 3:**
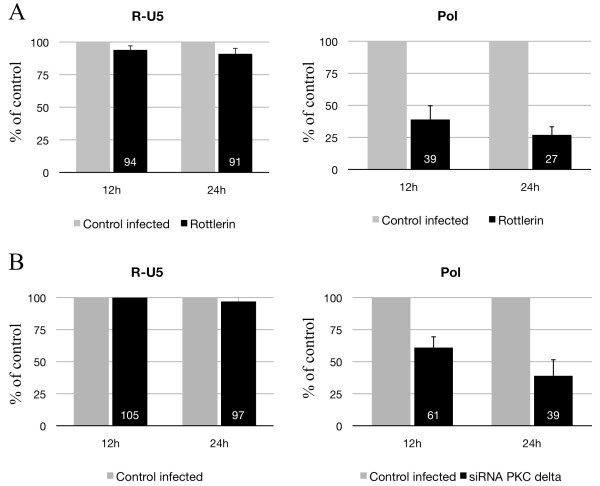
** Quantitative PCR of RT products (cDNA) from HIV-1 BaL-infected macrophages pretreated or not with rottlerin or accel siRNA against PKC-delta.** Macrophages (5x10^5^ cells/well) were pretreated by rottlerin (5 μM) for 30 minutes (**A**) or with accel siRNAs against PKC-delta for 3 days (**B**) and then infected with HIV-1 BaL (1 ng p24) for 3 h at 37°C and washed. Cells were then lysed at 12 h and 24 h after infection, and DNA was analyzed by PCR. Amplification of the early (R-U5) and late (pol) products of RT was performed by quantitative PCR (qPCR). Three independent experiments with macrophages from three different donors gave similar results.

Next, we analyzed early steps that follow the entry of HIV-1 into macrophages. To this end, we pretreated macrophages with rottlerin or siRNA against PKC delta and harvested viral DNA at different times after the infection (12 and 24 h). DNA was extracted and quantitative PCR analyses were conducted with oligonucleotides specific for early (R-U5) and late (pol) reverse transcription (RT) products (Figure 3). Early RT products were detected with all conditions (Figure 3). These results indicate that this early step of RT is not blocked following PKC-delta inhibition by rottlerin or knock-down by siRNA. In contrast, PCR amplification of late (pol) RT products was attenuated greatly after rottlerin treatment (Figure 3A). We observed a 61% and 73% inhibition at 12 h and 24 h, respectively. Late RT products were also reduced in siRNA-treated cells (Figure 3B). These results suggest that inhibiting PKC-delta inhibits the synthesis of late RT products in macrophages. Overall, these results suggest that PKC-delta is required at the level of early reverse transcription, soon after the initiation of viral cDNA synthesis.

### Inhibition of PKC-delta impairs the integrity of actin cytoskeleton in human macrophages

**Figure 4:**
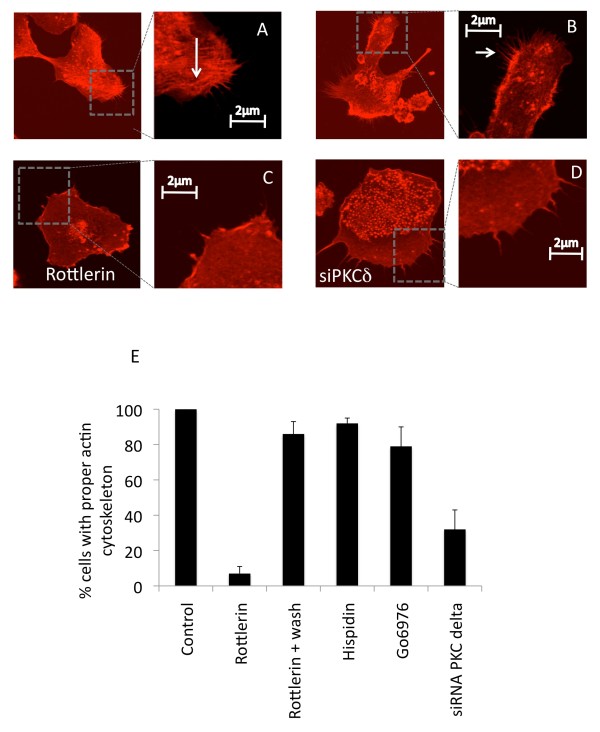
** Actin cytoskeleton is altered by pretreatment of macrophages by rottlerin (5 μM).** Human macrophages were treated for 24 h with rottlerin (5 μM, **C**), transfected with siRNAs to PKC-delta (**D**) or left untreated (**A** and **B**) and then fixed with formaldehyde (3.7%). Macrophages were then permeabilised with Triton X-100 (0.2%) and stained with rhodamine-phalloidin before mounting using moviol. Preparations were then observed by confocal microscopy. Confocal images are representative of three independent experiments. Arrows indicate stress fibers (**A**) and pseudopods (**B**). (**E**) Quantitative results represent the percentage of cells with proper actin cytoskeleton in the presence of different inhibitors or after treatment with siRNA against PKC-delta.

Since interaction between the RT complex and actin cytoskeleton is necessary for the elongation of reverse transcriptase [47], next we analyzed effects of rottlerin on the organization of actin cytoskeleton. Macrophages were preincubated with or without rottlerin for 24 h, and labeled with phalloidin, a specific ligand of F-actin (the monomer forming actin microfilaments), which was coupled to rhodamine, a fluorescent probe. Cells were then observed using confocal microscopy. As a control, untreated macrophages contained a number of pseudopods, which are projections of the cytoplasm towards the exterior of the cell that result from cytoskeletal rearrangements of actin (Figure 4A-B). In these untreated macrophages, actin microfilaments organize in stress fibers (Figure 4A, see arrow). However, in rottlerin-pretreated macrophages, very few pseudopods (Figures 4C) were observed, and they did not contain stress fibers (Figure 4C). Interestingly, this effect was reversible. Thus, in cells that were preincubated with rottlerin and then cultured without the inhibitor, we observed the restoration of normal cytoskeleton (Figure 4E lane 3). Importantly, siRNA against PKC-delta had similar effects on the actin cytoskeleton as rottlerin, although to a lesser extent (Figures 4D and 4E, lane 6). In addition, inhibitors of other PKC isozymes such as hispidin or go6976 had no major effects on actin filaments (Figure 4E). Thus, these data indicate that inhibiting PKC-delta affects the integrity of the actin cytoskeleton in macrophages.

**Figure 5:**
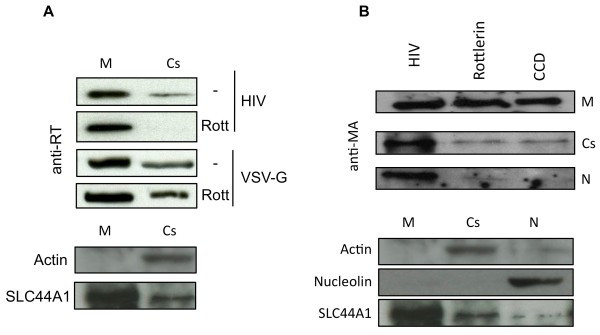
** Rottlerin reduces cytoskeletal association of HIV-1 RT complexes.** (**A**) Macrophages (5x10^6^) were left untreated or pretreated with rottlerin (Rott) and then infected with HIV-1 BaL (50 ng p24) or VSV-G-pseudotyped vector (10 ng p24). Membrane bound (M) and cytoskeletal (Cs) fractions were obtained and the presence of HIV-1 RT was assessed by Western blotting (upper panels). Purity of membrane bound and cytoskeletal fractions was analyzed by Western blotting for control proteins (bottom panels). (**B**) Macrophages (5x10^6^) were left untreated or pretreated with rottlerin or cytochalasin D (CCD) and then infected with HIV-1 BaL (50 ng p24). Membrane bound (M), cytoskeletal (Cs) and nuclear (N) fractions were obtained, and the presence of Gag-MA was assessed by Western blotting (upper panels). The purity of membrane bound, cytoskeletal and nuclear fractions was analyzed by Western blotting for control proteins (bottom panels).

Since the reverse-transcriptase complex from the incoming virus interacts with actin microfilaments [49], we hypothesized that inhibiting PKC-delta could lead to its dissociation from the actin cytoskeleton. To address this question, we fractionated cellular and cytoskeletal proteins from macrophages, which were pretreated or not with rottlerin and then infected with HIV-1 BaL. RT (Figure 5A) or matrix proteins (Gag-MA) (Figure 5B) were detected by Western blotting. In cells infected with HIV- or VSV-G-pseudotyped lentiviral vectors, RT was found in the membrane and cytoskeletal fractions (Figure 5A). However, RT was not found in the cytoskeletal fraction following the pre-treatment with rottlerin. Similar results were obtained using the Gag-MA as a marker (Figure 5B). Additionally, using cytochalasin D (CCD) as a control to disrupt actin polymerization, the Gag-MA was also not found in the cytoskeletal fraction. Taken together, these results suggest that PKC-delta is required for cytoskeletal integrity, which is essential for early steps in viral replication.

## Discussion

It was previously shown that PKC can be stimulated via interaction between CCR5 and gp120 [9]. This activation facilitates HIV-1 replication at different steps of its replicative cycle including entry, integration and gene expression [9,44,50,51]. However, these studies did not investigate thoroughly the role of different PKC isozymes in macrophages. For this reason, we investigated the involvement of PKC-delta, which plays an important role in the differentiation of macrophages, in HIV-1 replication. Our work was performed using complementary approaches including the chemical inhibitor rottlerin, specific antisense oligonucleotides, and specific siRNA. We demonstrated for the first time that HIV-1 is able to activate PKC-delta in macrophages. Importantly, we demonstrated that PKC-delta is critical for the replication of HIV-1 in human macrophages.

Several steps of the viral replicative cycle were analyzed to identify the one that was affected by this inhibition. Our results indicate that there is no block to viral entry upon inhibiting PKC-delta. Indeed, the expression of viral receptor (CD4) and co-receptor (CCR5) was not altered. Nevertheless, a recent study demonstrated that inhibiting PKC-alpha and/or beta could reduce the expression of these surface molecules in CD4 T lymphocytes [9]. It is thus possible that different PKC isozymes serve different functions in different cellular contexts. Further supporting our data, in the presence of PKC inhibitors, fusion occurred normally as assessed by syncytia formation in co-cultures with HeLa cells expressing R5/X4 and gp120/gp41 from HIV-1 Lai or HIV-1 ADA. This latter finding was confirmed by quantifying levels of intracellular p24 after incubating macrophages with HIV-1 ADA in the presence or absence of PKC inhibitors. All these studies, including levels of receptor/co-receptor and membrane fusion, suggest that the step of entry was not affected by inhibiting PKC-delta.

We also demonstrated that later steps, such as transcription, were not affected as demonstrated by the ability of Tat to activate the HIV-1 LTR similarly in the presence or absence of PKC inhibitors. This lack of effect of PKC-delta inhibitors on transcription was also confirmed with the expression of LTR-GFP from cells treated with rottlerin and transduced with VSV-G-pseudotyped vectors. Indeed, the transduction of macrophages with VSV-G-pseudotyped, but not with HIV-1 JR-FL lentiviral vectors, was insensitive to PKC-delta inhibition. VSV-G pseudotyped vectors use an alternative pathway for RTC (RT complexes) delivery to the cytosol and thus bypass HIV-mediated early entry steps. This difference of sensitivity to PKC-delta inhibitor thus indicates clearly that early steps of retroviral replicative cycle are the major targets of PKC-delta inhibition.

To analyze further, we used Q-PCR and demonstrated that the inhibition of PKC-delta affected a step prior to the first strand transfer, but following initiation of RT. Thus, the major step altered by PKC-delta inhibition occurs early in RT. Several reports revealed that the actin cytoskeleton of the target cell plays an important role in the early steps of replication [52]. Indeed, the interaction between RT complexes and actin is not only essential for efficient RT, but also for the transport of preintegration complexes to the nucleus [47,53,54]. Indeed, pretreatment of cells with cytochalasin D (CCD), an inhibitor of actin polymerization, prevents the infection by HIV-1. Because effects of PKC-delta inhibitors on HIV-1 replication appeared to occur at a post entry step, we also analyzed the actin cytoskeleton. Indeed, the C2 domain of PKC-delta contains an actin-binding site, which could be involved in the redistribution of actin in neutrophils [42,43]. Accordingly, we demonstrated that rottlerin and siRNA against PKC-delta altered the actin cytoskeleton in macrophages, which is in agreement with previous studies on PKC-delta.

Correlated to the impairment of the actin cytoskeleton, we demonstrated that RT and p17 Ma proteins in the incoming RT complex, which are used frequently as markers to monitor the RT complex, did not co-fractionate with the cytoskeleton when PKC-delta was inhibited. Indeed, several additional lines of evidence demonstrated a link between actin cytoskeleton and HIV-1 replication. First, a block at the level of early RT was previously reported using cytochalasin D (CCD), an inhibitor of actin cytoskeleton polymerization [47,55]. Second, viral particle-mediated induction of a signaling pathway via CXCR4 is required for infection of resting T cells [56-58]. In these cases, cofilin phosphorylates actin and participates in its redistribution, which overcomes the restriction related to cortical actin in resting T cells. Thirdly, Komano *et al*. demonstrated that inhibiting Arp 2/3, which is involved in actin polymerization, also restricts viral replication at an early stage in T cells [59]. Finally, Naghavi *et al*. implicated Moezin, which helps to tether cellular membranes to actin as being critical for early steps of viral replication [60]. Thus, our studies suggest that PKC-delta is a major signaling intermediary, which is activated by the virus to rearrange the actin network and thus facilitating early steps in the viral replicative cycle, particularly the RT step, in macrophages. Interestingly, recent studies have demonstrated the importance of a shallow endocytic pathway for HIV-1 entry and fusion [61]. Actin could thus play an important role in the completion of fusion after endocytosis. However, our VSV-G-pseudotyped vectors were not affected when PKC-delta was inhibited. Similar results were reported by Burkinskaya *et al*. who demonstrated that cytoskeletal impairment by CCD inhibits reverse transcription after entry of HIV-1, but not VSV-G pseudotyped vector. Thus, there is a difference between HIV- and VSV-G-mediated entries that requires PKC-delta and actin cytoskeleton integrity. It is possible that PKC-delta is critical only when fusion occurs independently of pH, while it is not required when fusion occurs under low pH conditions in late endosomes. It could i) favor fusion and uncoating which involve both viral and cellular factors, ii) allow transport of RTCs to permissive compartments containing cellular factors required for RT, and iii) trigger activation of RTCs by interaction with actin. However the exact mechanism remains to be clarified.

Of note, viral nucleocapsid and integrase also interact with actin [62,63]. Both of these proteins and Vpr are part of the incoming reverse transcriptase complex.

Macrophages are a major target of HIV-1 infection, due to their high levels of expression of CCR5 and their persistence in infected individuals. Macrophages are residents of different organs and tissues, including the central nervous system, and thus can be found in different microenvironments in which regular therapies may be less effective than in circulating CD4+ T cells. In these cells, pharmacokinetics and therapeutic efficiencies are understudied areas of research. Understanding better viral replication in macrophages could lead to the development of improved therapies in the future.

## Conclusions

This work shows that PKC-delta is activated following interaction between HIV-1 and human primary macrophages and plays a major role in viral replication. PKC-delta seems to play a role in early steps of the viral replicative cycle, allowing completion of reverse transcription. Our data suggest that this is due to a role of PKC-delta on the organization of proper actin cytoskeleton.

## Methods

### Cell culture

Peripheral blood mononuclear cells (PBMCs) were isolated from Buffy coats of healthy HIV-negative donors in a Ficoll density gradient (Pharmacia, Piscataway, NJ). PBMCs were then plated at a density of 10^6^ cells per well in 24-well Primaria (Becton Dickinson, Rutherford, NJ) tissue culture plates. Monocytes were isolated by adherence, after 45 minutes incubation in Iscove medium supplemented with human AB serum (10%). Monocytes were then washed 3 times with HBSS and cultivated during 7 days in Iscove medium supplemented with 10% Fetal Calf Serum (FCS), penicillin (100 units/ml) and streptomycin (100 pg/ml) at 37°C, 5% CO_2_, in a humid atmosphere so that macrophages can differentiate. M-CSF (10 ng/ml, Roche) was added on the first day of culture. Macrophages are 94% pure as tested by FACS with anti-CD14 antibody.

### Chemical inhibitors

Ro31-8220, a PKC inhibitor, rottlerin, a PKC-delta inhibitor, Hispidin, a PKC-beta inhibitor, Go6976, inhibitor of calcium dependent PKC izozymes alpha and beta1 and of PKCmu and cytochalasin D, an inhibitor of actin polymerization, have been obtained from Calbiochem.

### SiRNAs

Validated siRNA to human PKC-delta (Cat# sc-36253) [45,64] and control siRNA (Cat# sc-37007) were purchased from Santa Cruz Biotechnology (Santa Cruz, CA) and transfected in HeLa cells using siRNA transfection reagent from Santa Cruz Biotechnology (Cat# sc-29528).

Accel siRNAs to human PKC-delta (Cat# A-003524-16-0005) and control accel siRNA (Cat# D-001910-01-05) were purchased from Thermo scientific and introduced in human primary macrophages without transfection reagent, by simple incubation for 2 days before infection with HIV-1 BaL. Targetting sequence for siRNA to PKC-delta is GUUCUGUGCAAAGACUU.

The following phosphorothioate oligonucleotides sense GCCCCACCATGGCGCCGTTC and antisense GAACGGCGCCATGGTGGGGC (target region on mRNA PKC −8 to + 12) specific to PKC-delta were synthesized by Genset Oligos (Genset SA). Antisense oligonucleotides were previously assessed for their specificity [46] and used as previously described [46].

### Infection

HeLa-R5/X4 (2.10^5^ cells) were cultured in 12 well plates and transfected with siRNA control or siRNA PKC-delta (see above) using siRNA transfection reagent from Santa Cruz Biotechnology (Cat# sc-29528) at 10 or 30 nM. After 48 h, cells were infected with HIV-1 BaL or HIV-1 VN44 in DMEM 2% FCS and washed 2 times after 3 hours with DMEM. Cells were then cultivated in DMEM 10% FCS 1% PS. After 24 h, infection was scored via LTR-transactivation using X-gal coloration.

Macrophages (5.10^5^ cells) were cultured in 12 well plates and transfected with Accel siRNA control or Accel SiRNA PKC-delta at 10^-6^ M (Thermofisher, see above). After 48 h, cells were infected with HIV-1 BaL in DMEM 2% FCS and washed 2 times after 3 hours with DMEM. Macrophages were then cultivated in DMEM 10% FCS 1% PS. After 3 days, infection was assessed by detecting p24 in the supernatant using ELISA.

### Extraction of membrane and cytoplasmic proteins

After treatment of macrophages with HIV-1 BaL, 1 ng p24, macrophages (5.10^6^) were harvested at 30 minutes or 1 h and lysed at 4°C in 100 μl of hypotonic buffer A (20 mM Tris HCl, 2 mM EDTA, 1 mM DTT, 10 μg/ml leupeptin, 1 mM PMSF; pH 7,5) by repeated aspirations through a syringe fitted with a 21 Gauge needle. After the addition of 200 μl of fresh buffer B (20 mM Tris HCl, 2 mM EDTA, 1 mM DTT, 10 μg/ml leupeptine, 1 mM PMSF, 0.33 M sucrose; pH 7.5), the lysate was centrifuged at 100,000 g, 4°C, for 40 min. The supernatant, corresponding to the cytoplasmic fraction, was collected; proteins were quantified by the Bradford assay and stored at −20°C. The pellet, corresponding to the membrane fraction, was solubilised in 50 μl of fresh B buffer containing 1% of Triton X-100, sonicated (1 min, power 2.5), and the amount of proteins quantified and stored at −20°C.

### Extraction of total proteins

After macrophage treatment with HIV-1 BaL, 1 ng p24, during 30 minutes or 1 h, macrophages (2.10^6^) were harvested, centrifuged, and the pellet lysed in 200 μl of PBS 1% NP-40. The amount of proteins was quantified by the Bradford assay and then proteins were stored at −20°C.

### Extraction of cytoplasmic, membrane and cytoskeleton fractions

Macrophages (5.10^6^ cells) were lysed and cytoplasmic, membrane and cytoskeleton fractions obtained as previously described [47]. Anti-RT antibory is from abcam (ab63911) and anti-gagMA was obtained from the NIH reagents program.

### Western blotting

Identical amounts of proteins (10 μg) were separated on SDS-PAGE gel and then transferred to a nitrocellulose membrane. Immunoblotting was conducted by using either anti-PKC isozyme antibodies (anti-PKC-delta and anti PKC-beta_II_) at the 1:1000 dilution (Santa-Cruz biotechnology). Membranes were blocked in 5% milk, Tris-buffered saline, 0.05% Tween 20 (TTBS) for 1 h, washed 4 times with TTBS, and incubated with the primary antibody for 2 h. Immuno-reactive bands were detected by 2 h incubation with secondary antibodies directed against rabbit immunoglobulins conjugated with peroxydase (1:1000) (DAKO A/S, Roskilde, Denmark). Bands were visualized on film after incubation of the membranes with a chemiluminescent substrate (Pierce, Rockford, IL).

### Lentiviral vectors

293 T cells (2.5.10^6^) were cultured on a 150 mm Petri dish in DMEM 10% FCS, penicillin (100U/ml) and streptomycin (10 pg/ml), supplemented with L-glutamine (2 mM) for 24 h. Cells were then cotransfected by the phosphate calcium method, with 30 μg of gag-pol plasmid coding for capsid proteins and HIV-1 enzymes, 10 μg of plasmid coding for the VSV-G envelope, and 40 μg of lentiviral vector with GFP driven by CMV. The medium was changed 10 h after cotransfection. Supernatant was harvested 72 h after contransfection and centrifuged 15 minutes at 2500 rpm. Supernatant was filtered through a 0.22 μm filter and then ultracentrifuged 90 minutes at 50,000 g. The pellet was finally resuspended in 100 μl PBS.

### Transduction

Macrophages, isolated as previously described in 24 well plates, were preincubated with or without rottlerin during 2 hours and then incubated 3 h at 37°C in 250 μl of Iscove medium 2% FCS containing 50 μl of VSV-G/GFP vector per well, in presence or absence of inhibitors. After 2 washes with PBS, cells were cultivated in Iscove medium, 10% FCS. After 2 days, cells were visualized with a fluorescence microscope.

### Q-PCR

5 × 10^5^ macrophages/well in a 24-well plate were incubated for 3 h at 37°C in the presence of HIV-1 BaL virus (1 ng p24/ml) pretreated with DNase I. Cells were then washed with HBSS and Iscove medium, 10% FCS, 1% penicillin/streptomycin was added. Cells were then washed with HBSS at different times after infection. DNA was then extracted (Qiagen). For the detection of early (R-U5) and late (pol) reverse transcripts, DNA was amplified with the appropriate primers at 70°C in a LightCycler (Roche) with SYBR Green following the manufacturer’s recommendations. Viral DNA was normalized by cellular genomic GAPDH. Primers sequence: Strong-stop: (1) agcctgggagctctctggcta and (2) ccagagtcacacaacagacgg; Late: (1) and (3) cgcttcagcaagccgagtcct, GAPDH gene: (4) : ctctgacttcaacagcgac and (5) : tctctcttcctcttgtgctc

### Actin cytoskeleton analysis

Macrophages (5.10^5^ cells/well) were resuspended and placed in wells containing a glass slide. After two cycles of adherence, macrophages were washed 2 times with PBS, and then fixed with PBS medium 3.7% formaldehyde for 10 minutes at room temperature. After two more washes with PBS, macrophages were permeabilised by a 5 min incubation in the presence of 0.1% TRITON X-100. Two washes with PBS were performed, then cells were blocked with PBS 1% BSA for 30 min to avoid non-specific labeling. Cells were then labeled with phalloidine-rhodamine (1U/well/200 μl of PBS 1% BSA, Molecular Probes) for 20 minutes at room temperature. Macrophages were washed two more times with PBS and then mounted on cover slide using moviol and placed at 4°C until observation. Macrophages labeled with phalloidine-rhodamine were observed under a confocal microscope equipped with a 568 nm laser to excite the probe. 50 cells per slide were counted on at least 2 different slides per condition. Cells with clear pseudopodes were counted as positive while cells without pseudopodes or with small rare pseudopodes were negative. All results were normalized to control cells (100%).

### Syncytia formation

HeLa-R5/X4 (10000 cells) were cocultured with HeLa-gp120/gp41LAI or HeLa-gp-120/gp41ADA (10000 cells) in 96-well plates in the presence of various concentrations of each inhibitor. After 20 h, syncytia were scored by contrast phase microscopy.

## Competing interests

The authors declare that they have no competing interests.

## Authors’ contributions

XC and EB conceived and designed the experiments and analyzed the data. XC, OM and FG performed experiments. XC and EB wrote the paper. All authors read and approved the final manuscript.

## Supplementary Material

Additional file 1 **Figure S1.**(A) Macrophages were incubated with different chemical inhibitors (left panel) or increasing doses of rottlerin for 72h or DMSO as negative control. Cell viability was assessed by trypan blue staining. (B) Macrophages were incubated in the presence of sense or antisense oligonucleotides for 2 days and then infected with HIV-1 BaL (1ng p24) for 2 h. Macrophages were then washed and supernant collected after 72 h. P24 was quantified by ELISA and percentage of inhibition measured. (C) Western blot showing expression of PKC-delta in cell extracts from macrophages incubated with sense or antisense oligonucleotides for 48 h. As control, the effect of antisense PKC-delta was tested on the expression of PKC-alpha. In each case, antibodies specific to each PKC isoform were used in Western blot labeling. Click here for file

Additional file 2 **Figure S2.**HeLa CD4-CXCR4-CCR5 cells were left untreated or transfected with control siRNA (30nM) or siRNA to PKC-delta (10 or 30nM) and then infected with HIV-1 VN44 X4-tropic virus (1ng p24) for 3 h then washed. 48h later, cells were incubated in the presence of X-gal and infection was assessed by counting beta-gal positive cells by microscopy. Click here for file

Additional file 3 **Figure S3.**HeLa-R5/X4 cells with integrated LTR-beta-gal were preincubated or not with rottlerin (5μM) for 30 min and then left untreated or transduced using Gst-Tat (5μM). After 24 hours, beta-gal positive cells were scored using X-Gal by microscopy. LTR transactivation is relative and was set to 100% for Gst-Tat treated cells in absence of rottlerin. Click here for file
