# Supplementary figures and images for: Protein kinase C-delta regulates HIV-1 replication at an early post-entry step in macrophages
Source: Retrovirology. 2012 May 3;9:37. doi: 10.1186/1742-4690-9-37 (PMC3432598; doi:10.1186/1742-4690-9-37)

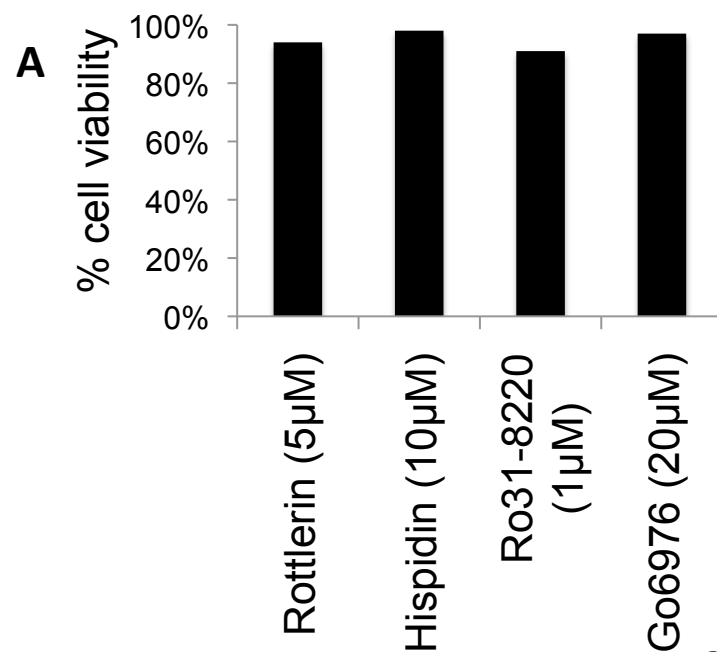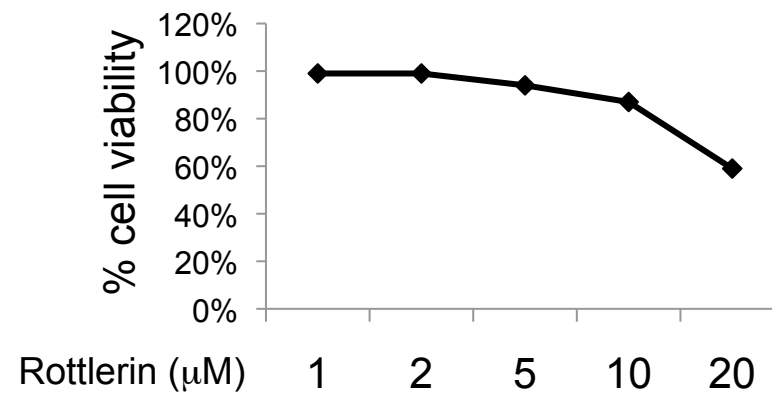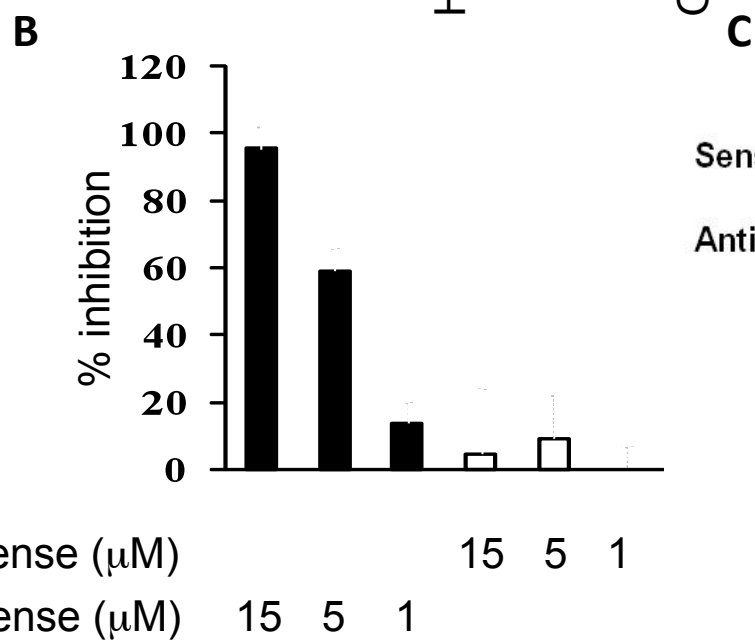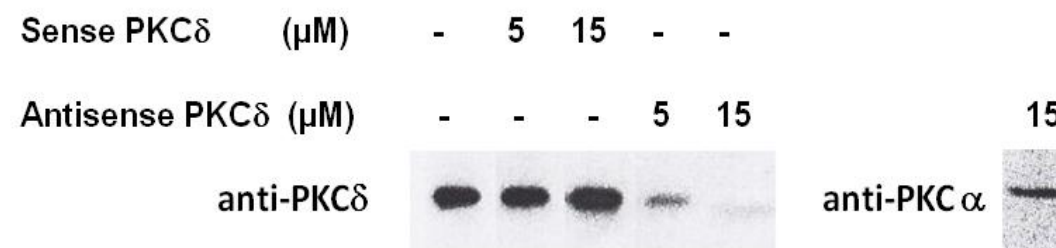

Supplementary figure 1

Supplement: Additional file 1 — Figure S1.(A) Macrophages were incubated with different chemical inhibitors (left panel) or increasing doses of rottlerin for 72h or DMSO as negative control. Cell viability was assessed by trypan blue staining. (B) Macrophages were incubated in the presence of sense or antisense oligonucleotides for 2 days and then infected with HIV-1 BaL (1ng p24) for 2 h. Macrophages were then washed and supernant collected after 72 h. P24 was quantified by ELISA and percentage of inhibition measured. (C) Western blot showing expression of PKC-delta in cell extracts from macrophages incubated with sense or antisense oligonucleotides for 48 h. As control, the effect of antisense PKC-delta was tested on the expression of PKC-alpha. In each case, antibodies specific to each PKC isoform were used in Western blot labeling. [file 1742-4690-9-37-S1.pdf]

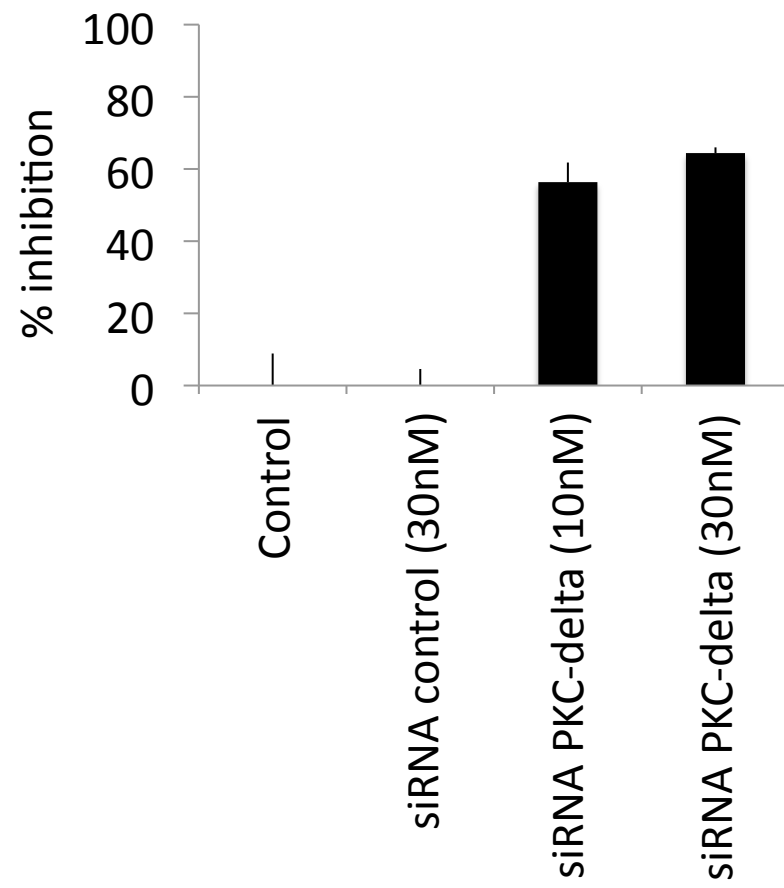

**Supplementary figure 2**

Supplement: Additional file 2 — Figure S2.HeLa CD4-CXCR4-CCR5 cells were left untreated or transfected with control siRNA (30nM) or siRNA to PKC-delta (10 or 30nM) and then infected with HIV-1 VN44 X4-tropic virus (1ng p24) for 3 h then washed. 48h later, cells were incubated in the presence of X-gal and infection was assessed by counting beta-gal positive cells by microscopy. [file 1742-4690-9-37-S2.pdf]

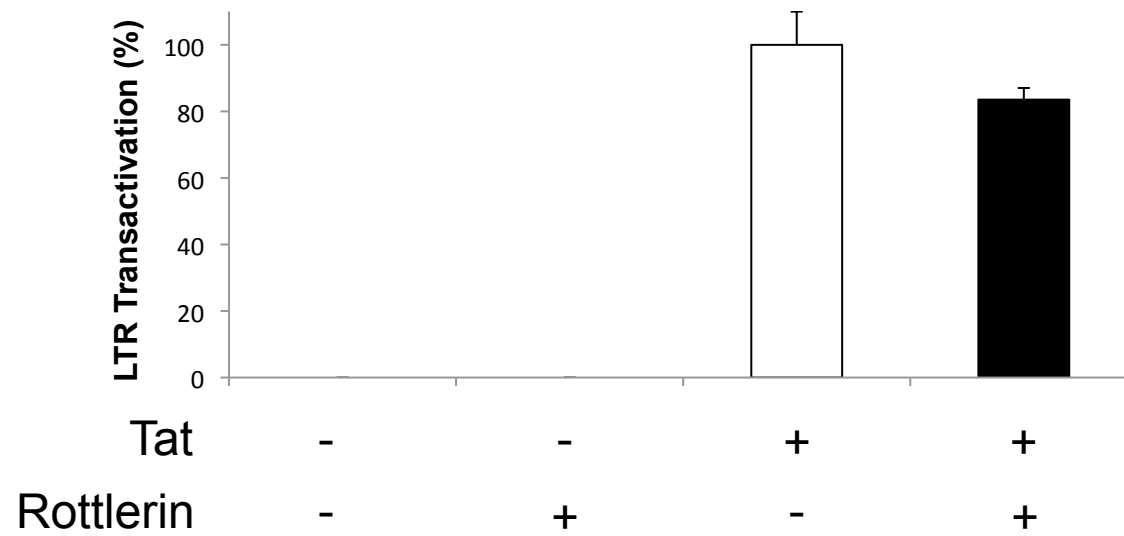

**Supplementary figure 3**

Supplement: Additional file 3 — Figure S3.HeLa-R5/X4 cells with integrated LTR-beta-gal were preincubated or not with rottlerin (5μM) for 30 min and then left untreated or transduced using Gst-Tat (5μM). After 24 hours, beta-gal positive cells were scored using X-Gal by microscopy. LTR transactivation is relative and was set to 100% for Gst-Tat treated cells in absence of rottlerin. [file 1742-4690-9-37-S3.pdf]
